# Association between Air Pollutants and Cardiovascular Disease Mortality in Wuhan, China

**DOI:** 10.3390/ijerph120403506

**Published:** 2015-03-25

**Authors:** Yisi Liu, Xi Chen, Shuqiong Huang, Liqiao Tian, Yuan’an Lu, Yan Mei, Meng Ren, Na Li, Li Liu, Hao Xiang

**Affiliations:** 1 School of Public Health, Wuhan University, Wuhan 430071, Hubei Province, China; E-Mails: roselewis@sina.com (Y.L.); aries_c_7@163.com (X.C.); 2012302170055@whu.edu.cn (M.R.); 2012302170047@whu.edu.cn (N.L.); 2 Global Health Institute, Wuhan University, Wuhan 430071, Hubei Province, China; 3 Hubei Provincial Center for Disease Control and Prevention, Wuhan 430079, Hubei Province, China; E-Mail: hsq7513@163.com; 4 State Key Laboratory of Information Engineering in Surveying, Mapping and Remote Sensing, Wuhan University, Wuhan 430079, China; E-Mail: tianliqiao@whu.edu.cn; 5 Environmental Health Laboratory, Department of Public Health Sciences, Univ Hawaii at Manoa, 1960 East West Rd., Biomed Bldg., D105, Honolulu, HI 96822, USA; E-Mail: yuanan@hawaii.edu; 6 School of Stomatology, Wuhan University, Wuhan 430071, Hubei Province, China; E-Mail: meiyan124@126.com; 7 Department of Epidemiology and Biostatistics, Guangdong Key Lab of Molecular Epidemiology, School of Public Health, Guangdong Pharmaceutical University, Guangzhou 510310, China; E-Mail: pupuliu919@gmail.com

**Keywords:** PM_10_, NO_2_, SO_2_, cardiovascular disease, mortality

## Abstract

We examined the associations of daily mean concentrations of ambient air pollutants (particulate matter (PM_10_), sulfur dioxide (SO_2_), nitric oxide (NO_2_)) and daily cardiovascular diseases (CVD) mortality in Wuhan, China using a case-crossover design to analyze four years of data (2006–2009) collected from the Hubei Provincial Center for Disease Control and Prevention and the Wuhan Environmental Protection Bureau. From 2006 to 2009, daily average concentrations of PM_10_, SO_2_ and NO_2_ were 115.60 µg/m^3^, 53.21 µg/m^3^ and 53.08 µg/m^3^, respectively. After adjusting for temperature and relative humidity, a 10 µg/m^3^ increase in SO_2_ and NO_2_ over a 24-h period was associated with CVD mortality relative risk (R.R.) of 1.010 (95% CI: 1.000, 1.020) for SO_2_ and 1.019 (95% CI: 1.005, 1.033) for NO_2_, but there was no significant association between increases in PM_10_ and mortality. Subgroup analysis on by gender showed a significant association of 1.026 (95% CI: 1.007, 1.045) between NO_2_ and CVD among males, while no significant statistical effect was shown among females. Subgroup analysis by age showed that for those older than 65 years, every 10 µg/m^3^ increase in NO_2_ was associated with a 1.6% (95% CI: 0.1%, 3.1%) increase in CVD mortality. Subgroup analysis on different types of CVD showed that every 10 µg/m^3^ increase in PM_10_ and SO_2_ were significantly associated with an approximately 1.012 (95% CI: 1.002, 1.022) and 1.021 (95% CI: 1.002, 1.040) increase, respectively, in ischemic heart disease (ICH) mortality. In conclusion, exposure to NO_2_ is significantly associated with CVD mortality. Larger, multi-center studies in Chinese cities are being currently conducted to validate these findings.

## 1. Introduction

In recent years, more and more evidence has shown that particulate matter (PM_10_), sulfur dioxide (SO_2_), nitric oxide (NO_2_), and other major air pollutants have acute adverse effects on the human circulatory system. Zeka *et al*. [1] analyzed data from 20 U.S. cities between 1989 and 2000 and found a statistically significant association between air particulate matter PM_10_ and heart disease mortality. Katsouyanni *et al*.’s study on 12 European cities [2] and Samoli *et al*.’s study on 30 European cities [3] showed that the elevated concentrations of SO_2_ and NO_2_ were associated with cardiovascular disease (CVD) mortality. Meanwhile, a study conducted by the Chinese scholars Yang and Pan [4] showed that, in addition to SO_2_ and NO_2_, PM_10_ is also associated with CVD mortality. However, different pollutant concentration levels and lifestyles in the various regions studied produce inconsistent findings. Qian and his co-workers [5] showed that daily concentrations of PM_10_ in Wuhan were associated with residents’ CVD mortality. However, since this study was conducted from 2001 to 2004, and because air pollutant levels have changed substantially in recent years, this data may no longer be representative of Wuhan’s present air pollution and related CVD conditions. In our study, a case-crossover design was used to determine the link between changes in air pollutant concentration and CVD mortality in Wuhan, China.

## 2. Materials and Methods

### 2.1. Data Source

Meteorological data was collected from the Wuhan Meteorological Station monitoring database. This data included the daily average temperature and relative humidity from 1 January 2006 to 31 December 2009.Atmospheric pollutant data for daily air pollutants were released by the Municipal Environmental Protection Bureau of Wuhan, including the average daily Air Quality Index (AQI) of air pollutants PM_10_, NO_2_, SO_2_ from 2006 to 2009, and the above information was converted into a daily average concentration of air pollutants based on the “Ambient Air Quality Standard” (GB3095-2012) [6]. According to the breakpoints of air pollutant and the AQI and air pollutant concentration conversion formula [7], we converted the AQI values to concentration levels using the standard formula [8].

Death data was collected from the Hubei Provincial Center for Disease Control and Prevention death registration system, which included information of decedents from 1 January 2006 to 31 December 2009. According to “the International Classification of Diseases, ICD-10”, a few common cardiovascular diseases, including ischemic heart disease (I20–I25), essential hypertension (I10) and stroke (I60–I69), were analyzed for any association with air pollution.

### 2.2. Methods

#### Ethics Statement

All subjects gave their informed consent for inclusion before they participated in the study. The study was conducted in accordance with the Declaration of Helsinki, and the protocol was approved by the Ethics Committee of Wuhan University (No.13003).

Case-crossover studies are used in epidemiology to study the effects of exposures or risk factors that are short-term or transient. Each research subject consists of two data components: the “case” period, the timeframe during which the subject is considered a case; and the “control” period, the timeframe during which the subject is considered as a control (*i.e*., not a case, prior to exposure). Within each individual, the effects of exposure during the case period are compared with those during the control period; therefore, the two components are “matched pairs”, and each subject serves is its own control.

After adjusting for daily average temperature and relative humidity, the present study analyzed the relative risk (RR.) value of association of air pollutant exposure (until death) during the case period and during the control period using the case-crossover design and conditional Logistic regression. For all analyses, the statistical software packages SAS (version 9.2) and C-CAT were used, the lag effect of the increase in air pollutant concentration was taken into consideration, and meteorological factors were introduced as covariates into the regression model for the association analysis.

## 3. Results

### 3.1. Information on Air Pollutants and Meteorological Factors

**Figure 1:**
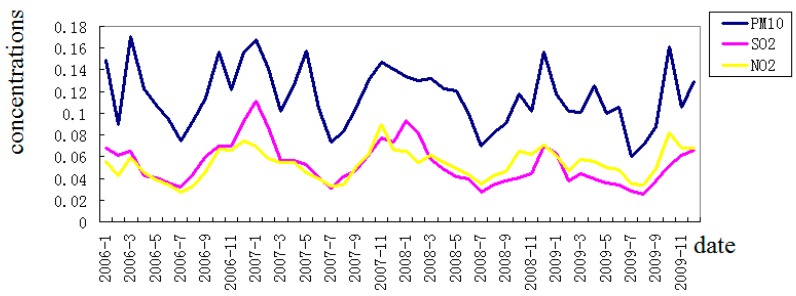
Monthly average concentrations (μg/m^3^) of air pollutants during 2006–2009.

From 1 January 2006 to 31 December 2009, there were 22,489 deaths (excluding accidents and injuries) due to disease in Wuhan, 8,955 of which were related to CVD. Among all subjects, 53.6% were males while 46.4% were females, and subjects who were equal or older than 65 years old represented 80.1% (Table 1). During the same time period, the daily average concentrations of PM_10_, SO_2_ and NO_2_ were 115.60 μg/m^3^, 53.21 μg/m^3^ and 53.08 μg/m^3^, respectively (Table 2). Daily average concentrations of the three pollutants showed considerable seasonal variation in that they were higher during spring and winter than during summer and autumn (Figure 1).

**Table 1:** Description of characteristics of all subjects collected during 2006–2009.

Characteristics		2006 (%)	2007 (%)	2008 (%)	2009 (%)	Total (%)
Gender	Male	399 (63.2)	1153 (52.8)	1306 (53.4)	1944 (52.6)	4802 (53.6)
	Female	232 (36.8)	1031 (47.2)	1138 (46.6)	1752 (47.4)	4153 (46.4)
Age Group	<65	207 (32.8)	427 (19.6)	472 (19.3)	674 (18.2)	1780 (19.9)
	≥65	424 (67.2)	1757 (80.4)	1972 (80.7)	3022 (81.8)	7175 (80.1)
Total		631	2184	2444	3696	8955

**Table 2:** Descriptive analysis of air pollutants and meteorological factors in Wuhan, 2006–2009.

Air Pollutants and Meteorological Factors	Daily Avg.	SD	Percentile					IR
			0	25	50	75	100	
PM_10_ (µg/m^3^)	115.60	54.59	18.00	74.00	108.00	148.00	567.00	74.00
SO_2_ (µg/m^3^)	53.21	29.91	8.00	31.00	48.00	68.00	267.00	37.00
NO_2_ (µg/m^3^)	53.08	21.51	17.60	36.80	49.60	65.60	153.60	28.80
Avg. Temperature (°C)	18.14	9.49	−2.70	9.40	19.70	26.20	35.30	16.80
Avg. humidity	0.70	0.13	0.21	0.62	0.71	0.79	0.97	0.17

### 3.2. Correlations between Air Pollutant Concentrations and Meteorological Factors

The daily average concentrations of the air pollutants PM_10_, SO_2_ and NO_2_ were shown to be significantly associated with each other, as shown by the rank correlation coefficients 0.6164 (*p* < 0.01), 0.7005 (*p* < 0.01), and 0.6962 (*p* < 0.01), respectively. All three air pollutant concentrations were negatively correlated with the meteorological factors temperature and humidity, with the correlations of PM_10_ to temperature and humidity being weaker in comparison with the correlations of SO_2_ and NO_2_ to the same meteorological factors (Table 3).

**Table 3:** Spearman rank correlation coefficient analysis of meteorological factors and daily concentrations of air pollutants in Wuhan, 2006–2009.

Air Pollutants and Meteorological Factors	PM_10_	SO_2_	NO_2_	Avg. Temperature	Avg. Humidity
PM_10_	1				
SO_2_	0.6164	1			
NO_2_	0.7005	0.6962	1		
Avg. temperature	−0.1775	−0.4060	−0.3345	1	
Avg. humidity	−0.1803	−0.1974	−0.106	−0.1834	1

### 3.3. Association of PM_10_, SO_2_ and NO_2_ with Daily CVD Mortality

After adjusting for average daily temperature and relative humidity, the results showed that the associations between the concentrations of NO_2_, SO_2_, and CVD mortality were most statistically significant on the same day, and also the next day, but the association was no longer statistically significant two days later. Every 10 μg/m^3^ increase in NO_2_ was associated with a 1.9% (95% CI: 0.5%, 3.3%) increase in daily CVD mortality after 24 h and a 1.7% (95% CI: 0.4%, 3.1%) increase after 48 hours. In addition, each 10 μg/m^3^ increase in SO_2_ daily concentration were both significantly associated with 1.0% (95% CI: 0.0%, 2.0%) increases in daily CVD mortality after 24 and 48 h, respectively. PM_10_ levels were shown to not have any significant association with daily CVD mortality; for every 10 μg/m^3^ increase in PM_10_ concentration, the RR value was 1.005 (95% CI: 1.000, 1.010) (Table 4).

### 3.4. Subgroup Analysis of Associations between PM10, SO2, NO2 and Daily CVD Mortality

The associations among female CVD mortality and PM_10_, SO_2_, and NO_2_ were not significant. As for males, while the associations among PM_10_, SO_2_ and male daily CVD mortality were not statistically significant, each 10 μg/m^3^ increase in NO_2_ was shown to be significantly associated with 2.6% (95% CI: 0.7%, 4.5%) and 2.5% (95% CI: 0.6%, 4.4%) increases in CVD mortality after 24 h and 48 h, respectively (Table 3). Upon age stratification, the associations among PM_10_, SO_2_ and daily CVD mortality of people over the age of 65 were not statistically significant; however, every 10 μg/m^3^ increase in NO_2_ was significantly associated with a 1.6% (95% CI: 0.1%, 3.1%) increase in CVD mortality for the people over the age of 65 (Table 4).

Analysis was also done on the association of essential hypertension (I10), ischemic heart disease (IHD; I20–I25), and stroke (I60–I69) with the same three air pollutant concentrations. Results showed that each 10μg/m^3^ increase of PM_10_ was significantly associated with a 1.2% (95% CI: 0.2%, 2.2%) increase in IHD mortality. Every 10μg/m^3^ increase in SO_2_, was significantly associated with a 2.1% (95% CI: 0.2%, 4.0%) increase in IHD mortality, and was most significant after 24 h. NO_2_, however, did not seem to be significantly associated with IHD mortality. None of the pollutants analyzed seemed to have any statistically significant associations with essential hypertension and stroke (Table 4).

**Table 4:** Associations between air pollutants and CVD mortality among urban residents in Wuhan.

Categories of Death	LAG days	PM_10_		NO_2_		SO_2_	
		H.R. (95% CI)	*p* Value	H.R. (95% CI)	*p* Value	H.R. (95% CI)	*p* Value
Total death of CVD (8955)	0	1.005 (1.000, 1.01)	0.0673	1.019 (1.005, 1.033)	0.0068	1.010 (1.000, 1.020)	0.0472
	1	1.004 (0.999, 1.009)	0.0842	1.017 (1.004, 1.031)	0.0130	1.010 (1.001, 1.020)	0.0354
	2	0.999 (0.994, 1.003)	0.5775	0.994 (0.981, 1.008)	0.4000	0.997 (0.988, 1.007)	0.5506
	Avg. 3 days	1.002 (0.995, 1.008)	0.6317	1.010 (0.992, 1.028)	0.2780	1.007 (0.994, 1.020)	0.3125
Male death of CVD (4802)	0	1.006 (0.999, 1.013)	0.0929	1.026 (1.007, 1.045)	0.0066	1.008 (0.994, 1.022)	0.2638
	1	1.007 (1.000, 1.014)	0.0573	1.025 (1.006, 1.044)	0.0090	1.012 (0.999, 1.025)	0.0798
	2	0.996 (0.989, 1.002)	0.2100	0.990 (0.972, 1.008)	0.2640	0.989 (0.976, 1.002)	0.1037
	Avg. 3 days	1.001 (0.992, 1.010)	0.8368	1.014 (0.990, 1.039)	0.2536	1.002 (0.985, 1.021)	0.7883
≥65 death of CVD (7175)	0 1 2 Avg. 3 days	1.003 (0.997, 1.009) 1.003 (0.998, 1.009) 0.998 (0.993, 1.003) 1.000 (0.992, 1.007)	0.3458 0.2694 0.5019 0.9429	1.016 (1.001, 1.031) 1.013 (0.998, 1.029) 0.992 (0.978, 1.007) 1.005 (0.985, 1.025)	0.0385 0.0945 0.3229 0.6478	1.009 (0.998, 1.020) 1.009 (0.998, 1.020) 0.997 (0.987, 1.008) 1.006 (0.991, 1.020)	0.1087 0.1023
							0.5957 0.4378
Death of IHD (I20–I25) (2519）	0 1 2 Avg. 3days	1.012 (1.002, 1.022) 1.007 (0.998, 1.017) 0.994 (0.985, 1.003) 1.006 (0.995, 1.018)	0.0147 0.1394 0.2019 0.2886	1.023 (0.997, 1.049) 1.012 (0.987, 1.038) 0.990 (0.965, 1.015) 1.012 (0.981, 1.045)	0.0781 0.3586	1.013 (0.995, 1.032 1.021 (1.002, 1.040)	0.1644 0.0261
					0.4336 0.4389	1.001 (0.984, 1.020) 1.019 (0.996, 1.043)	0.8770 0.1136

## 4. Discussion and Conclusions

The results of this study showed that among the three air pollutants analyzed, the associations between NO_2_, SO_2_, and daily CVD mortality were statistically significant, but no association between PM_10_ and CVD mortality was found. For the subgroup analysis, no significant associations were found between air pollutants and CVD mortality among females, while significant associations were found for NO_2_ among men as well as among people of ages 65 and older; upon cause-specific analysis, significant associations were found between PM_10_, SO_2_, and ischemic heart disease mortality, while no significant associations were found for essential hypertension and stroke.

In the *Ambient Air Quality of Key Environmental Protected cities in the First Half of 2012* report of the Ministry of Environmental Protection, Wuhan met secondary ambient air quality standards, and the average concentrations of PM_10_, SO_2_, NO_2_ decreased annually. Of the three major pollutants, PM_10_ was the most abundant in Wuhan, with the highest daily average concentration. The average concentration of SO_2_ was significantly lower than that in Urumqi (92.4 μg/m^3^) [9], which is likely due to the relatively lower amount of coal-burning done in Wuhan. NO_2_ in Wuhan was lower than in Beijing [10] and Guangzhou [11], likely due to less vehicle ownership among Wuhan residents compared to that in Beijing and Guangzhou, thus lowering the amount of automobile emission released into the atmosphere. The average concentration of PM_10_ was significantly lower in Wuhan compared to that in Xi’an [12], which is likely due to the fact that Xi’an is a very industrial city. Although, compared with Shanghai, which also reached secondary ambient air quality standards, air pollutants concentrations in Wuhan were all higher [13]. Thus, there is room for improvement of the air quality in Wuhan.

The results of this study demonstrated that increases in NO_2_ and SO_2_ are significantly associated with daily CVD mortality, while no statistically significant associations were found for PM_10_ (*p* > 0.05). These findings were consistent with results of Song *et al*.’s study [14] done in Shanghai as well as Venners *et al*.’s study [15] done in Chongqing. A study by Deng *et al*. [16] showed that each 10 μg/m^3^ increase in PM_10_, increased the risk associated with circulatory system diseases, cardiovascular disease, cerebrovascular disease mortality by 0.36% (95% CI: −0.07, 0.78), 0.63% (95% CI: −0.02, 1.28) and 0.33% (95% CI: −0.26, 0.92), respectively. Some studies were found to contrast the present study’s findings. For example, Qian *et al*. [5] found that PM_10_ concentration was associated with CVD mortality, each 10 μg/m^3^ increase in PM_10_ daily concentration at lag 0 day was significantly associated with an increase in cardiovascular 0.51% (95% CI: 0.28,0.75). Wang *et al*. [17] pointed out in their meta-analysis that the conclusions of associations between acute effects from short-term exposure to PM_10_ and population CVD mortality were invalid due to the following factors: (1) limitations of study subjects and sample size; (2) lack of adjustment of weather factors or other atmospheric confounding pollutants; and (3) incomplete adjustments of particulate matters’ acute effects on the cardiovascular system. Also, we speculate the following additional factors may explain the differences between the present study and others: (1) the variation in particulate matter components and toxicity across different areas; (2) the negligible risk of CVD mortality caused by PM_10_; and (3) considerable differences in sample size across these studies.

According to the gender-stratified analysis, no statistically significant associations were found between pollutants and CVD mortality in females, and among males, only NO_2_ was significantly associated with CVD mortality. In contrast, research conducted by Kan *et al*. [18] in Shanghai suggested that women were more susceptible to air pollution exposure, and that this exposure-mortality association was related to the level of education. Also, a study by Kunzl *et al*. [19] indicated that effects of air pollution on non-smokers were greater in comparison to the effects on smokers. Therefore, it is possible that different educational levels and smoking prevalence across various regions may contribute to differences among genders.

In the age-stratified analysis, NO_2_ was associated with daily CVD mortality in people over 65 years of age, which is consistent with the results of a study done by Yang *et al*. [20] Also, as McGrath [21] pointed out, more elderly people suffer from chronic diseases than any other age group, likely making them more susceptible to the adverse effects of air pollution exposure. Therefore, health conditions characteristic of elderly populations could explain the differences observed across age groups in this study.

As for the cause-specific analysis, only PM_10_ and SO_2_ were significantly associated with IHD. The association of PM_10_ and SO_2_ with IHD mortality obtained in this study was consistent with a study done by Milojevic *et al*. [22] In addition, studies done by Zeka *et al*. [1] and Wong *et al*. [23] also showed that PM_10_ and SO_2_ were associated with IHD mortality. Baskurt *et al*. [24] found that SO_2_ could generate denatured methemoglobin leading to reduced permeability of red blood cells, and thus a lack of blood supply to the organs. Meanwhile, PM_10_ could alter the generations of vascular tone, atherosclerosis, autonomic nervous effects, and systemic inflammatory response [25], which may further explain the observed increases in IHD mortality related to SO_2_ and PM_10_ concentrations. While no associations were found between the pollutants and primary hypertension and stroke in the present study, other studies found that PM_10_ and NO_2_ are actually associated significantly with stroke [5,26]. For instance, Qian found every 10 μg/m^3^ increase in PM_10_ daily concentration at lag 0 day was significantly associated with an increase in stroke by 0.44% (95% CI: 0.16,0.72). Yang *et al*. [27] pointed out that because cardiovascular diseases are caused by a combination of multiple gene and environmental risk factors, the epidemiological characteristics of CVD vary in different regions, period and populations. Thus, inconsistencies across different research studies is expected.

In this study, daily CVD mortality was generally associated with NO_2_, and this may be because the main source of atmospheric NO_2_ pollution was automobile emission. The average annual growth of vehicle holdings in Wuhan was 23%, and air pollutions caused by urban traffic was on the rise, further increasing the NO_2_-related CVD mortality, a cycle which has also been found in other regions abroad [28,29]. As the primary air pollutant in Wuhan, PM_10_ showed a downward trend of concentration levels in recent years; however, compared to trends of SO_2_, NO_2_ concentrations, it was relatively higher. PM_10_ continues to be the primary air pollutant in Wuhan, having effects on CVD mortality similar to the past. As for PM_10_, this study did not find any significant association with daily population CVD mortality, perhaps due to the adsorption composition and toxicity unique to Wuhan.

This study used a case-crossover design for statistical analysis. Compared to the widely used time-series study methodology, case-crossover studies rely on a rational design rather than statistical models for controlling many potential confounders. They also reduce the bias of selecting control groups and save sample size through self-contrast. Stratification with respect to gender, age, and cause was also conducted for the analyses. Thus, results reflect the adverse health effects of air pollutants on different groups of people.

There were some limitations in this study. This study only involved single pollutant models instead of multi-pollutant model analysis. From a statistical point of view, results obtained when using single pollutant models differ from those obtained when using multi-pollutant models. Air pollutants are a mixture of PM_10_, SO_2_, NO_2_, and a variety of other pollutants. Therefore, further research is needed to identify which type of model is more suitable to explain air pollution, and which gives results more representative of a population’s health status. In addition, considering air conditioner usage, personal lifestyles, and other factors, the actual exposure of air pollutants to the population and levels of air pollutant concentrations are relatively independent; thus, it is likely that exposure measurement errors are present in this study. It was also difficult to estimate the individual exposure. We suggest that individual and geographical differences should be considered in extrapolation of the presented conclusions. Some studies showed the synthetic actions of meteorological conditions and air pollutants [30], but this study did not collect data on factors that may have had an effect on results, such as atmospheric pressure, wind direction, wind speed, precipitation, *etc.* In addition, for case-crossover design, time-varying factors are possible confounders-e.g., day of week and long term trends, and results from the design are relative risks but not absolute risks. However, in summary this study shows that there is an association between exposure to air pollution and CVD mortality in Wuhan, China.
